# Untranslated regions (UTRs) are a potential novel source of neoantigens for personalised immunotherapy

**DOI:** 10.3389/fimmu.2024.1347542

**Published:** 2024-03-15

**Authors:** Christopher C. T. Sng, Ashwin Adrian Kallor, Benjamin S. Simpson, Georges Bedran, Javier Alfaro, Kevin Litchfield

**Affiliations:** ^1^ Cancer Research UK Lung Cancer Centre of Excellence, University College London (UCL) Cancer Institute, London, United Kingdom; ^2^ International Center for Cancer Vaccine Science, University of Gdansk, Gdansk, Poland; ^3^ Department of Biochemistry and Microbiology, University of Victoria, Victoria, BC, Canada; ^4^ Institute for Adaptive and Neural Computation, School of Informatics, University of Edinburgh, Edinburgh, United Kingdom

**Keywords:** UTR, untranslated region, neoantigen, checkpoint inhibitor, personalised vaccine, PrimeCUTR, immunopeptidomics

## Abstract

**Background:**

Neoantigens, mutated tumour-specific antigens, are key targets of anti-tumour immunity during checkpoint inhibitor (CPI) treatment. Their identification is fundamental to designing neoantigen-directed therapy. Non-canonical neoantigens arising from the untranslated regions (UTR) of the genome are an overlooked source of immunogenic neoantigens. Here, we describe the landscape of UTR-derived neoantigens and release a computational tool, PrimeCUTR, to predict UTR neoantigens generated by start-gain and stop-loss mutations.

**Methods:**

We applied PrimeCUTR to a whole genome sequencing dataset of pre-treatment tumour samples from CPI-treated patients (n = 341). Cancer immunopeptidomic datasets were interrogated to identify MHC class I presentation of UTR neoantigens.

**Results:**

Start-gain neoantigens were predicted in 72.7% of patients, while stop-loss mutations were found in 19.3% of patients. While UTR neoantigens only accounted 2.6% of total predicted neoantigen burden, they contributed 12.4% of neoantigens with high dissimilarity to self-proteome. More start-gain neoantigens were found in CPI responders, but this relationship was not significant when correcting for tumour mutational burden. While most UTR neoantigens are private, we identified two recurrent start-gain mutations in melanoma. Using immunopeptidomic datasets, we identify two distinct MHC class I-presented UTR neoantigens: one from a recurrent start-gain mutation in melanoma, and one private to Jurkat cells.

**Conclusion:**

PrimeCUTR is a novel tool which complements existing neoantigen discovery approaches and has potential to increase the detection yield of neoantigens in personalised therapeutics, particularly for neoantigens with high dissimilarity to self. Further studies are warranted to confirm the expression and immunogenicity of UTR neoantigens.

## 1 Introduction

Neoantigens arise from mutated proteins which can be processed and expressed on the surface of cancer cells, forming key targets in anti-tumour immunity. The success of checkpoint inhibitor (CPI) immunotherapy, particularly in tumours with a high mutational burden (as a proxy of neoantigen load), has spurred interest in the identification of the underlying neoantigens (1, 2). Early studies found neoantigens predicted from somatic mutations could stimulate patient-derived CD8^+^ T-cells, and were associated with response to CPI treatment (3–6). Furthermore, small studies demonstrating the ability of neoantigen-specific T cells to induce tumour regression hinted at the promise of neoantigen-directed therapy (7–9). More recently, neoantigen vaccine trials have demonstrated vaccine-induced T cell expansion, and evidence of durable disease response in some patients (10–13). Thus, these cancer-specific antigens represent an important target in development of personalised immunotherapy.

Traditional approaches to neoantigen identification have typically involved sequencing the protein-coding regions of the cancer genome for missense or insertion-deletion mutations, followed by HLA-binding prediction and neoantigen prioritisation (14). This may yield hundreds to thousands of putative neoantigens, but only a small fraction appear to contribute to immune responses (6, 7, 15). In a combined effort, the Tumour Neoantigen Selection Alliance (TESLA) global consortium identified 608 top-ranked neoantigens in 6 solid cancer samples, of which only 37 (6%) could be recognised by matched patient T cells (16). Likewise, approaches to identify predicted neoantigens in cancer immunopeptidomes have had limited yield (6, 17). Additionally, certain tumour types such as neuroblastoma and pancreatic adenocarcinoma bear an inherently low mutational burden, reducing the pool of candidate neoantigens (18). Further obstacles to immune recognition include immune-exclusion, immunosuppressive microenvironment, variable gene expression, mRNA quality control pathways (e.g. nonsense-mediated decay), and intra-tumoural heterogeneity (14, 19). To date, no personalised neoantigen-directed therapies have emulated the clinical response rates or widespread regulatory approval of CPI treatment.

**Figure 1:**
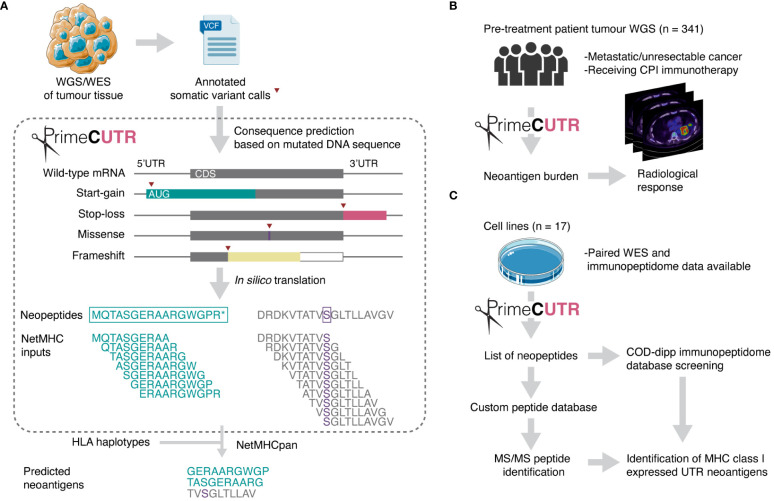
Schematic overview of this study. **(A)** PrimeCUTR accepts annotated somatic variant calls in Variant Call Format (VCF) files and returns an output of predicted neopeptides which can be used for downstream MHC class I binding assessment (netMHC inputs). Two example neopeptides are shown (left – start-gain, right – missense) depicting the sliding window processing step to generate 10-mer netMHC inputs. CDS - coding sequence. **(B)** UTR neoantigens were predicted in a whole genome sequencing (WGS) dataset and assessed for relationship with radiological response to CPI therapy. **(C)** Cell line whole exome sequencing (WES) data was obtained for prediction and identification of MHC class I- presented UTR neoantigens. Neopeptides were screened against COD-dipp, a database of mass spectrometry (MS)-identified canonical and non-canonical MHC class I antigens (24). Tumour and petri dish illustration adapted from Servier https://smart.servier.com/ and licensed under CC-BY 3.0. Radiological response icon is adapted from images courtesy of Bruno Di Muzio, Radiopaedia.org, rID: 65164.

Given the attrition of candidate neoantigens through the discovery process, expanded neoantigen search strategies are essential to capture the breadth of neoantigens to direct therapeutic design. Various studies have demonstrated the presentation on MHC class I of non-canonical/cryptic peptides arising from ostensibly non-coding regions or alternative reading frames (20–23). The majority of these studies focus on non-mutated peptides which are not necessarily cancer-specific, increasing the likelihood self-tolerance. In this study, we present a novel R package, PrimeCUTR, which identifies candidate neopeptides in the 5’ and 3’ untranslated region (UTR) of genes generated by premature start-gain and stop-loss mutations respectively. Start-gain mutations create novel open reading frames (neoORFs) through the generation of novel upstream start-codons (uAUG) within the 5’UTR region of an mRNA transcript (**Figure 1A**). Meanwhile, stop-loss mutations which convert the stop codon into a sense codon, theoretically result in read-through of the 3’UTR following the canonical peptide sequence. We describe how these neoantigens contribute to the immune landscape of cancer. To our knowledge, this is the first publicly available tool to predict these UTR neoantigens.

## 2 Methods

### 2.1 Cohort

355 patients with metastatic cancer who received CPI treatment were selected from the Hartwig Medical Cohort for analysis of pre-treatment somatic tumour mutation calls in conjunction with clinical response data. Of these, 341 patients had HLA typing data available and were included in this study. This cohort consisted of patients with melanoma (n = 153), lung cancer (n = 69), bladder cancer (n = 58), renal cancer (n = 20) and other cancers (n = 41). Corresponding whole genome sequencing (WGS) somatic mutation data was obtained in Variant Call Format (VCF) via Hartwig Medical Foundation data access request (license agreement DR-087). These VCFs files were generated by the Hartwig Medical Foundation and received aligned to GRCh37 (25). Variants were filtered to include only those with a PASS flag. Based on RECIST1.1 criteria treatment response data, patients with a complete or partial response were classified as CPI responders while those with stable or progressive disease were classified as CPI non-responders. While stringent, this grouping is concordant with published immunotherapy biomarker validation studies (26, 27). Median neoantigen burden was compared between responding and non-responding groups using the Wilcoxon signed-rank test. Multivariable logistic regression was used to assess this relationship while correcting for tumour mutational burden (TMB). TMB was defined as number of mutations per megabase (mut/MB).

### 2.2 Identifying start-gain and stop-loss neoantigens

Start-gain mutations were defined as any single-nucleotide variant (SNV) or short insertion-deletion which resulted in a new ATG codon in the upstream 5’UTR region of a transcript (uAUG). All uAUG-forming mutations in 5’UTR sequences from all Ensembl-annotated protein coding transcripts were included in the neoantigen prediction. The relevant reference genome (GRCh37 or GRCh38) was used depending on the prior alignment of the somatic mutation calls. Reading 5’ to 3’, in silico translation of the cDNA sequence, beginning from the uAUG was performed until a stop codon (TAA, TAG or TGA) was reached. Stop-loss mutations were defined as any SNV which altered the annotated stop codon of a transcript into a sense codon. In this case, in silico translation is continued from the new sense codon until a stop codon is reached. Open reading frames from insertion-deletion mutations were obtained similarly according to the preceding reading frame. In rare cases where no stop codon is reached within the transcript, the alternate reading frame (from a start-gain, stop-loss or insertion-deletion mutation) is read through to the mRNA poly-A tail, resulting in a poly-lysine sequence (28). Protein-coding SNVs, dinucleotide variants (DNVs), and small in-frame insertion-deletions are grouped as missense mutations. In this report, missense and frameshift mutations refer specifically to mutations occurring in the protein-coding regions of the genome.

All neopeptides were processed using a sliding window to generate 9-, 10- and 11-mers which included at least one mutated/frameshifted residue (**Figure 1A**). These peptides were then assessed for predicted MHC class I binding strength, using the pVACtools suite (version 3.1.1) to run pVACbind with the netMHCpan algorithm (29, 30). Peptides binding with an IC50 of less than 500 nM were reported as neoantigens. This widely adopted threshold is consistent with previous work showing that most MHC class I ligands bind below this affinity (31, 32). Start-gain neoORFs overlapping in-frame with coding sequences of other isoforms were excluded by removing any reported neoantigens with exact matches in the canonical human proteome.

### 2.3 Neoantigen dissimilarity

Neoantigen dissimilarity from the self-proteome (dissimilarity score) and neoantigen homology to known immunogenic epitopes from Immune Epitope Database (foreignness score) were calculated using the *foreignness_score* and *dissimilarity_score* functions in antigen.garnish 2 (https://github.com/andrewrech/antigen.garnish accessed 16th October 2023, (33)) with default parameters. Neoantigens were considered highly dissimilar based on a dissimilarity score of >0.7, and highly foreign with a cut-off foreignness score of >0.75. These cutoffs were selected based on natural breaks in the distribution of scores (**Supplementary Figures 1B, C**).

### 2.4 Mutational signature extraction

Mutational signatures were extracted for each tumour sample using DeconstructSigs (34) yielding the relative contribution of mutational processes per tumour sample (COSMIC v.1.0) (35). To obtain a score per cancer group, samples were grouped by type and the relative contribution of each mutational signature was averaged across samples. COSMIC mutational profiles (v1.0) were downloaded from https://cancer.sanger.ac.uk/signatures/downloads/ (accessed 16^th^ September 2023). To assess the probability of start-gain formation for a given mutational signature, all non-overlapping 5’UTR sequences in the human genome were obtained from Ensembl (GRCh38 Ensembl release 109). For each given COSMIC mutational signature profile, the probability of each unique single base substitution (SBS) was multiplied by the number of 5’UTR sites where such a substitution lead to uAUG formation, divided by the total number of 5’UTR sites where the substitution could occur. The probabilities of start-gain formation for each of the 96 SBS were then summed to give a probability of start-gain formation for each mutational signature.

### 2.5 Mass spectrometry (MS) validation

We identified 13 cell lines with whole exome sequencing (WES) data in the Cancer Cell Line Encyclopedia (CCLE, https://depmap.org/portal/download/all/ accessed 24^th^ May 2023) which had paired immunopeptidomic sequencing (**Supplementary Table 1A**). CCLE somatic mutations were downloaded aligned to GRCh38. Somatic mutation calls, aligned to GRCh37, from 4 melanoma patient samples were obtained from Bassani-Sternberg et al. (6). PrimeCUTR was used to predict start-gain, stop-loss and frameshift neopeptides from the somatic mutation calls of each of these 17 tumour/cell line samples. In the first validation step, neoORF peptides were compared to a non-canonical MHC-associated peptide database (Closed Open *De novo* – deep immunopeptidomics pipeline (COD-dipp)) generated from MS analysis (24). As a second validation step, Fragpipe version 19.1 (36) was used to performed independent proteogenomic MS database search by appending the neopeptides to the normal protein database as described previously (24). Ion, PSM and peptide-level false discovery rates were set at 1%.

### 2.6 Translation initiation site prediction

Web-based TIS prediction algorithms, TISRover (37, http://bioit2.irc.ugent.be/rover/tisrover) and TIS Transformer (38, https://jdcla.ugent.be/) were used to assess the likelihood of translation initiation of start-gain neoORFs. The cDNA sequence for the mutant transcripts were uploaded in FASTA format for analysis using default settings.

## 3 Results

### 3.1 Inferring UTR neoantigens from cancer mutation data

PrimeCUTR accepts somatic mutation VCF data annotated by Ensembl Variant Effects Predictor (VEP) (39) or the Hartwig Medical Foundation variant calling pipeline (25) (**Figure 1A**). VEP annotates mutation consequence per given gene transcript, providing necessary information for PrimeCUTR to classify mutations into missense, frameshift, 5’UTR variant and stop-loss. All 5’UTR variants are checked for start-gain formation (as of v111, VEP does not annotate start-gain variants), while all SNVs arising in the final codon of protein coding transcripts are additionally checked for stop-loss regardless of VEP annotation. The *get.peptide* function can be used interactively in R to predict the resulting neopeptide from pairs of Ensembl transcript ID and Human Genome Variation Society (HGVS) coding DNA sequence variant nomenclature provided by VEP. The *get.orfs* function accepts whole VCF files producing three outputs: (1) tab-separated files containing neopeptides per mutation class per sample (see **Supplementary Tables 1B–E** for example output), (2) FASTA-format text files containing 9, 10 and 11-mers which include at least one mutated/frameshifted residue, (3) relevant log files. *get.orfs* also returns neopeptides with normal flanking residues extending to the next up- or downstream trypsin cleavage site with or without a missed cleavage, allowing seamless integration of PrimeCUTR output into a proteomic search pipeline. The 9,10 and 11-mer FASTA files can be fed directly to MHC-binding prediction algorithms for neoantigen prediction. Additionally, for start-gain mutations, *get.peptide* scores start-gain Kozak consensus sequence strength (weak, moderate or strong – see Whiffin et al. (40)), estimates overlap with wild-type open reading frames, and flags potential in-frame overlap with protein coding sequences, allowing rapid screening of the most promising neopeptides. Further details on installation, usage and output of the PrimeCUTR R package, including a tutorial and example datasets can be found at https://github.com/christophersng/primeCUTR.

### 3.2 Incidence of UTR neoantigens

**Figure 2:**
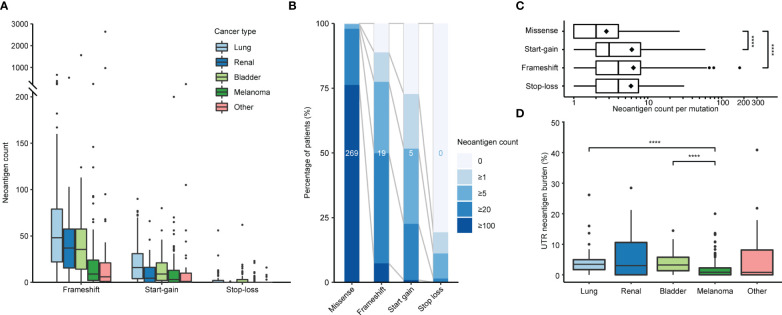
Incidence of neoantigens by class and cancer type. **(A)** Predicted frameshift, start-gain and stop-loss neoantigen count by cancer type. **(B)** Relative incidence of neoantigens across the patient cohort binned by neoantigen count. Values in the middle of each bar represent the median neoantigen count per patient. **(C)** Number of neoantigens generated per mutation segregated by class. Diamonds indicate mean values. **(D)** Proportion of UTR neoantigens by cancer type with pairwise comparison using Wilcoxon Rank Sum tests with Benjamini-Hochberg correction. Only significant values are indicated in plots **(C, D)**. ****, *p* ≤ 0.0001.

We applied PrimeCUTR and netMHCpan to pre-treatment cancer WGS samples from the Hartwig Medical Foundation to identify the contribution of the different mutation classes to the neoantigen landscape in patients who received CPI treatment (n = 341) (**Figure 1B**). Across the cancer types, the majority of predicted neoantigens with MHC binding (IC50 < 500 nM) arose from missense mutations (SNVs, DNVs, in-frame indels, total 177670, 88.8%), while frameshift, start-gain and stop loss mutations contributed 17254 (8.6%), 4701 (2.3%) and 563 (0.3%) respectively (**Table 1**). Among cancer types, lung cancer had the highest burden of mutations and neoantigens in all mutation classes (**Figure 2A**). Start-gain neoantigens were predicted in 72.7% of patients across cancer types, with a median of 5 unique neoantigens per patient (range 0-237) while stop-loss neoantigens were predicted in only 19.3% of patients (**Figure 2B**). By comparison, frameshift mutations were predicted in 88.9% of patients. Overall, frameshift, start-gain and stop-loss mutations generated neoORFs of similar lengths (median: 19 versus 20 versus 17 amino acid residues respectively). Although rarer, start-gain mutations generated significantly more neoantigens per mutation than missense mutations (median: 3 versus 2, mean: 6.09 versus 2.76, adjusted *p*-value < 2×10^-16^) (**Table 1** and **Figure 2C**).

**Table 1:** Predicted neoantigens by mutation class.

Class	Mutations (n)	Neoantigens (n)	Neoantigens per mutation	High foreignness neoantigens (n)*	High dissimilarity neoantigens (n)*
Missense	64424	177670	2.76	16438 (9.3%)	1137 (0.6%)
Frameshift	2694	17254	6.40	2155 (12.5%)	1302 (7.5%)
Start-gain	772	4701	6.09	542 (11.5%)	310 (6.6%)
Stop-loss	95	563	5.93	56 (10.0%)	34 (6.0%)

*Percentages expressed as a proportion of total neoantigens in each given mutation class.

Given the translation of neoORFs, we hypothesised start-gain and stop-loss mutations would be more distinct from the self-proteome. Previously, Richman et al. (33) demonstrated that neoantigens with high dissimilarity from the self-proteome, as well as high homology to known immunogenic peptides from Immune Epitope Database (foreignness score) were correlated with measures of immunogenicity. Using the same approach (see Methods), we found that frameshift, start-gain and stop-loss neoantigens were approximately 10 times more likely to have high dissimilarity compared to missense mutations (**Table 1**). Therefore, across the cohort, while UTR neoantigens only accounted for 2.6% of predicted neoantigens, they comprised 12.4% of high dissimilarity neoantigens (**Supplementary Figure 1A**).

Most UTR neoORFs were private: Only 2 start-gain mutations and no stop-loss mutations were shared by 3 or more patients. The two recurrent start-gain mutations occurred exclusively in melanoma samples: RPL8; ENST00000262584:c.-94G>A (7 patients, 4.6%) and DCAF7; ENST00000310827:c.-207G>A (3 patients, 2.0%). They respectively produced neoORFs 54 and 58 amino acid residues long, and were predicted to generate multiple patient-specific HLA binding neoantigens (**Supplementary Tables 2A–C**). These recurrent mutations were identified in an independent melanoma WGS cohort (41), where 6 patient samples (3.3%) had RPL8; ENST00000262584:c.-94G>A and 1 patient sample had DCAF7; ENST00000310827:c.-207G>A. Neither variant was reported in dbSNP, The Cancer Genome Atlas or gnomAD.

### 3.3 Start-gain incidence by mutational signature

All neoantigen classes were correlated with TMB (**Supplementary Figure 2A**). Interestingly, despite having a median TMB comparable to lung cancer (16.2 versus 16.4 mut/MB, **Supplementary Figure 2B**), melanoma showed significantly lower relative UTR neoantigen burden (0.8%) compared to lung (3.5%; corrected *p*-value = 4.9×10^-8^) and bladder (3.2%, corrected *p*-value = 4.8×10^-6^) malignancies (**Figure 2D**). This primarily reflected the lower relative incidence of start-gain mutations in melanoma.

**Figure 3:**
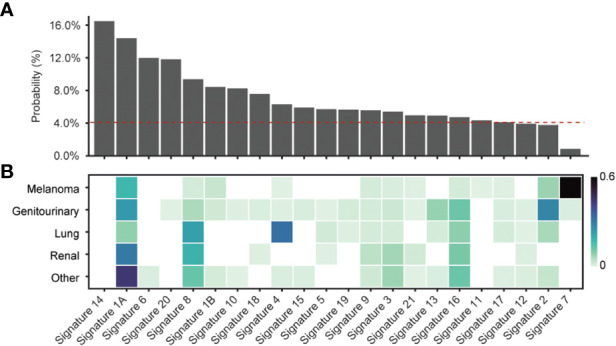
**(A)** Probability of start-gain formation based on COSMIC v1 mutational signature. The red dashed line indicates the probability of start-gain formation given a neutral mutational signature. **(B)** Heatmap of relative composition of mutations attributable to a given mutational signature averaged within each cancer group. Proposed aetiologies for the mutational signatures include: aging (1A/B), smoking (4), DNA mismatch repair (6, 14, 15, 20, 21), POLE mutation (10,14) and UV (7) (35, 42).

Given the majority of start-gain mutations arose from SNVs generating a new AUG codon in the 5’UTR, we hypothesised that the underlying single base substitution (SBS) mutational signature could explain the differences in relative start-gain mutation frequency between cancer types. We aggregated the probabilities of uAUG formation in all unique 5’UTR sequences of the human genome for every given 96 SBS mutational signature (COSMIC v1, 35) (**Figure 3**). This showed that mutational signatures linked to aging (1A/B), smoking (4), DNA mismatch repair (6, 14, 15, 20, 21) and POLE mutation (10, 14) favoured the formation of start-gain mutations (42). Meanwhile, ultraviolet (UV)-related mutational signature 7 strongly suppressed the likelihood of start-gain formation, explaining the relative sparsity of start-gain neoantigens in melanoma.

### 3.4 Response to checkpoint inhibitor immunotherapy

As described above, UTR neoantigens generate proportionally more neoantigens with a high dissimilarity from the self-proteome and thus may be a more potent immune target. We therefore assessed whether UTR neoantigen load was associated with response to CPI treatment. In univariate analysis, CPI responders had significantly higher missense and start-gain neoantigens (**Supplementary Figure 3**). Missense mutations are closely linked to TMB, an established marker of CPI response (2). The significant association of start-gain neoantigens and CPI response was not maintained in multivariable logistic regression when accounting for TMB (*p*-value = 0.8).

### 3.5 Immunopeptidomic discovery

**Figure 4:**
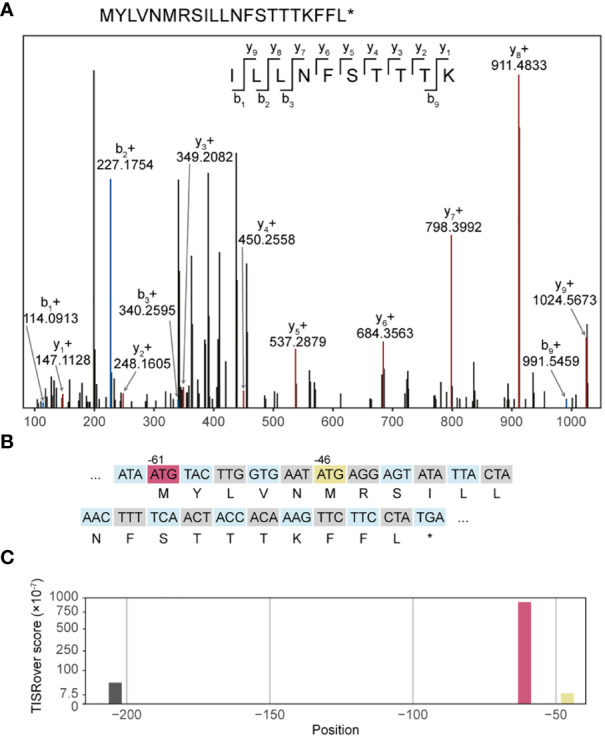
Immunopeptidomic discovery of a start-gain neoantigen. **(A)** Representative MS2 spectrum of ILLNFSTTTK. **(B)** Representation of codons in the mutated 5’UTR sequence in transcript ENST00000539214 containing the start-gain c.-61C>T (red), as well as the predicted neopeptide (second row). A wild-type uAUG is also highlighted (yellow). **(C)** Visualisation of TISRover output from the 5’UTR section in which each bar represents a TISRover score for a uAUG.

In order to demonstrate the expression of UTR neoantigens on MHC class I, we screened the UTR neopeptides against COD-dipp, a database of MS-identified canonical and non-canonical MHC class I antigens (24). 48 UTR neoORFs were predicted in 12 out of 17 studied cell lines from somatic mutation data (**Supplementary Table 1A**). Among these, one candidate UTR neoantigen, peptide ILLNFSTTTK, was identified in COD-dipp, matching a neoORF from a private start-gain mutation (OAT; ENST00000539214:c.-61C>T) in the Jurkat cell line (**Supplementary Table 1D**, row 40). This was further confirmed with high-confidence using independent proteogenomic MS database search of two Jurkat immunopeptidome replicates (**Figure 4A**). ILLNFSTTTK was identified in 7 and 8 different spectra in each replicate respectively, but not in any other cell line nor in the normal immunopeptidome. Of the known HLA alleles for Jurkat (43), MHC-binding prediction with netMHCpan showed that ILLNFSTTTK binds strongly to HLA-A*03:01 (17.13 nM). However, we noted that ILLNFSTTTK is also overlapped by a wild-type upstream open reading frame (uORF) beginning at position -46 (**Figure 4B**). While the wild-type uORF is not previously described in a repository of wild-type RIBO-Seq identified uORFs (www.sorfs.org, accessed 26^th^ April 2023) (44), the origin of ILLNFSTTTK from this uORF could not be excluded. To investigate the origin of translation of ILLNFSTTTK, we used TISRover to compare the likelihood of translation of the wild-type and mutant uORF (37). TISRover appeared to favour the mutant start-gain uORF (score: 9.2×10^-5^) over the wild-type uORF (score: 1×10^-6^) as a translation initiation site (TIS), although both scored lower than the canonical start codon (score: 2.8×10^-2^) (**Figure 4C** and **Supplementary Table 1F**). One study used a TISRover score cut-off of 0.1 to annotate translation initiation sites (45). Another TIS prediction algorithm, TIS Transformer, did not annotate either the wild-type or mutant uORFs as potential TIS (38).

Separately, we searched for the presence of the recurrent start-gain neoORFs RPL8; ENST00000262584:c.-94G>A and DCAF7; ENST00000310827:c.-207G>A within melanoma immunopeptidome datasets in the COD-dipp database. This identified peptide SAALVNRTR, which matched the RPL8; ENST00000262584:c.-94G>A neoORF, exclusively in the immunopeptidome of one patient-derived melanoma within all three replicate samples. SAALVNRTR was found to have strong patient-specific HLA binding to HLA-A*68:01 (**Supplementary Table 2D**). No peptides corresponding to RPL8; ENST00000262584:c.-94G>A were found in 10 healthy skin immunopeptidome datasets. SAALVNRTR had also been predicted from genomic data in our primary patient cohort (**Supplementary Table 2B**). In contrast to ILLNFSTTTK, this neoORF had no overlap with wild-type uORFs or coding regions. TISRover and TIS Transformer both verified the mutant uORF as a viable translation initiation site (**Supplementary Tables 2E, F**).

Taken together, this evidence supports the translation and expression of the start-gain neoORFs in a tumour-specific manner.

## 4 Discussion

In this study, we present PrimeCUTR, an open-source R package to identify UTR start-gain and stop-loss neopeptides from tumour somatic mutation calls. PrimeCUTR is applicable to WGS data as well as WES data (albeit limited by UTR coverage). PrimeCUTR is easily incorporated into any bioinformatic neoantigen discovery workflow and is scalable to the processing of large datasets via a high-performance computing cluster.

Using PrimeCUTR, we show that UTR neoORFs occur frequently across different subtypes of cancer, yielding a previously overlooked source of neoantigens. Like frameshift mutations, when compared to missense mutations, start-gain and stop-loss mutations yield more than double the neoantigens per given mutation. We show that start-gain mutation frequency is influenced by background mutational signature, being favoured in MMR deficiency (Signatures 6, 14, 15, 20 and 21) or POLE mutations (Signatures 10 and 14) which can be found in colorectal and endometrial cancers, as well as age (Signature 1A/B) and tobacco smoking (Signature 4) (42). Signature 8, of unconfirmed aetiology but previously observed in cancers including breast cancer, is also associated with start-gain mutation formation. Meanwhile, UV exposure (Signature 7) suppresses the formation of start-gain mutations in melanoma, although this is offset by the higher overall mutational burden in melanoma. Prevailing mutational signatures for a given cancer type may therefore guide whether personalised neoantigen profiling strategies should use extended UTR coverage sequencing approaches.

Previous studies have demonstrated that neoantigen dissimilarity from the self-proteome is an important predictor of immunogenicity and immunoediting (33, 46). In our analysis, frameshift and UTR neoantigens were found to be more dissimilar to the self-proteome than missense neoantigens and thus enriched amongst the pool of highly dissimilar neoantigens. As the cognate T cells of UTR neoantigens are less likely to have been subject to central mechanisms of tolerance, they represent a promising target for boosting anti-tumour immunity, while minimising off-target effects.

Studying 17 cell lines with paired somatic mutation and immunopeptidomic data, we identified one MHC class I-presented UTR neoantigen, ILLNFSTTTK, which matched a predicted private start-gain neoORF (OAT; ENST00000539214:c.-61C>T) in the Jurkat cell line. This peptide, along with its associated start-gain mutation was exclusive to Jurkat cells. This paired-discovery approach was limited by the fact that the cell line mutation data was derived from WES. Commonly used WES kits only cover up to 20% of UTR bases (47). Taking a more general approach, we searched for expression of recurrent UTR neoantigens within large immunopeptidomic datasets. From our primary WGS patient cohort, we identified a recurrent start-gain neoORF (RPL8; ENST00000262584:c.-94G>A) in 7 patients with melanoma, which was validated in 6 patient samples from an independent melanoma WGS cohort (41). This identified a further UTR neoantigen, SAALVNRTR, in one patient which exactly matched the RPL8; ENST00000262584:c.-94G>A neoORF (**Supplementary Table 2D**).

Current MS approaches detect only a small fraction of expressed peptides, compounding our limited identification of UTR neoantigens. Cuevas et al. (48) found that only 0.44% of non-canonical translation events (including uORFs within the 5’UTR) were detected by MS. Nevertheless, this is, to our knowledge, the first immunopeptidomic discovery of start-gain UTR neoantigens.

In the patient cohort, we found that UTR neoantigen burden was not significantly associated with CPI response when correcting for TMB. However, the significant disparity between predicted neoantigens and those able to elicit immune responses (6, 7, 15–17) suggests CPI response is driven by a small but important fraction of neoantigens. Prioritisation of these neoantigens for therapy remains a technical and biological challenge: beyond MHC class I binding affinity and dissimilarity, factors such as intratumoural heterogeneity, RNA expression, and location within the protein sequence all influence expression and immunogenicity (14, 49). The expression of uORFs (and start-gain mutations) is also governed by the sequence context of the translation initiation site. Given the current limitations of immunopeptidomic validation, incorporation of translation initiation prediction algorithms will be critical to support the prioritisation of UTR neoantigens (37, 38). While our computational approach has yielded two promising candidates for expressed UTR neoantigens, validation of MS spectra with synthetic peptides was not done due to limited access to the identical MS instrumentation utilised across the diverse source datasets. Further studies with paired whole genome and immunopeptidomic analysis of patient tumour samples, as well as T cell reactivity assays are ultimately needed to confirm the expression and immunogenic potential of UTR neoantigens. In summary, we describe a computational tool to study the contribution of UTR neoantigens to the immune landscape of cancer with the potential to boost neoantigen search strategies for personalised immunotherapy.

## Data availability statement

The data analysed in this study is subject to the following licenses/restrictions: Patient level data from the Hartwig Medical Foundation is considered private identifiable information and therefore access-controlled. Requests to access these datasets should be directed to https://www.hartwigmedicalfoundation.nl/en/data/data-access-request/.

## Ethics statement

Ethical approval was not required for the study involving humans in accordance with the local legislation and institutional requirements. Written informed consent to participate in this study was not required from the participants or the participants’ legal guardians/next of kin in accordance with the national legislation and the institutional requirements. All patients provided explicit consent to the Hartwig Medical Foundation for data sharing for cancer research in accordance with the license agreement.

## Author contributions

CS: Conceptualization, Data curation, Formal analysis, Investigation, Methodology, Project administration, Software, Validation, Visualization, Writing – original draft, Writing – review & editing. AK: Conceptualization, Formal analysis, Investigation, Methodology, Software, Writing – review & editing, Visualization. BS: Conceptualization, Formal analysis, Investigation, Methodology, Software, Writing – review & editing, Data curation, Writing – original draft. GB: Conceptualization, Formal analysis, Investigation, Methodology, Software, Visualization, Writing – review & editing. JA: Conceptualization, Resources, Supervision, Writing – review & editing. KL: Conceptualization, Funding acquisition, Methodology, Project administration, Resources, Supervision, Writing – review & editing.

## Supplementary material

Supplementary Figure 1
**(A)** Proportion of neoantigens originating from different mutation classes, stratified by neoantigen quality metrics. High binding neoantigens were those with predicted IC50 <50nM. Histogram of **(B)** foreignness scores and **(C)** dissimilarity scores for all neoantigens. High foreignness (>0.75) and high dissimilarity thresholds (>0.7) indicated by red bars.

Supplementary Figure 2
**(A)** Correlation matrix of neoantigen count and tumour mutational burden (TMB). **(B)** TMB values by cancer type.

Supplementary Figure 3 Univariate analysis of CPI response based on **(A)** missense, **(B)** frameshift, **(C)** start-gain or **(D)** stop-loss neoantigen count.
