# Supplementary figures and images for: Untranslated regions (UTRs) are a potential novel source of neoantigens for personalised immunotherapy
Source: Front Immunol. 2024 Mar 15;15:1347542. doi: 10.3389/fimmu.2024.1347542 (PMC10978585; doi:10.3389/fimmu.2024.1347542)

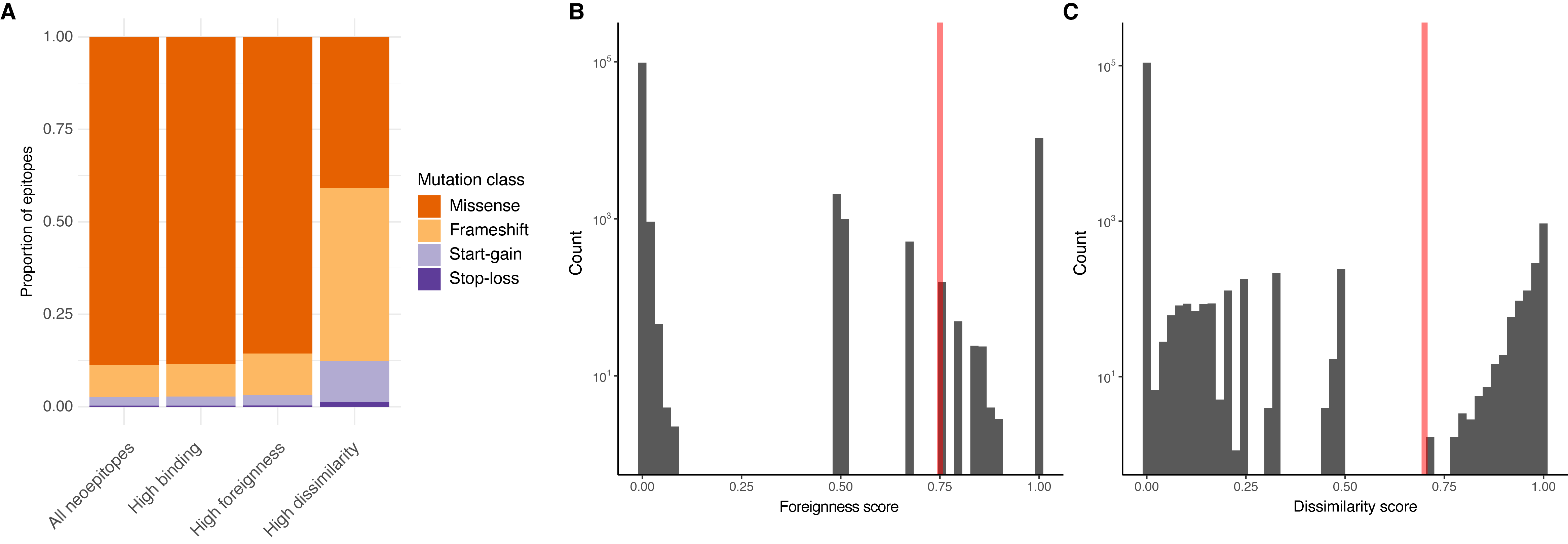

Supplement: Supplementary Figure 1 — (A) Proportion of neoantigens originating from different mutation classes, stratified by neoantigen quality metrics. High binding neoantigens were those with predicted IC50 <50nM. Histogram of (B) foreignness scores and (C) dissimilarity scores for all neoantigens. High foreignness (>0.75) and high dissimilarity thresholds (>0.7) indicated by red bars. [file Image_1.tif]

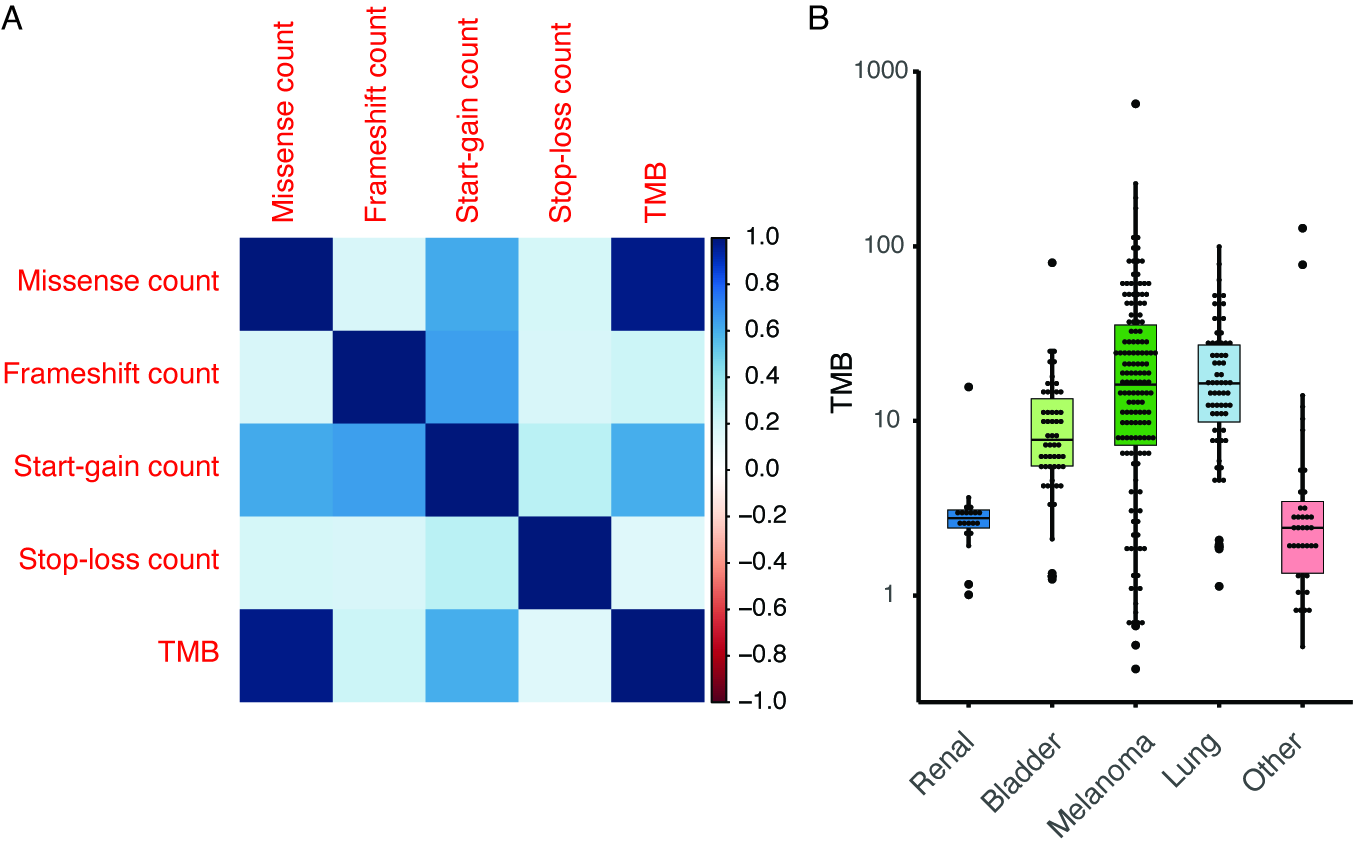

Supplement: Supplementary Figure 2 — (A) Correlation matrix of neoantigen count and tumour mutational burden (TMB). (B) TMB values by cancer type. [file Image_2.tif]

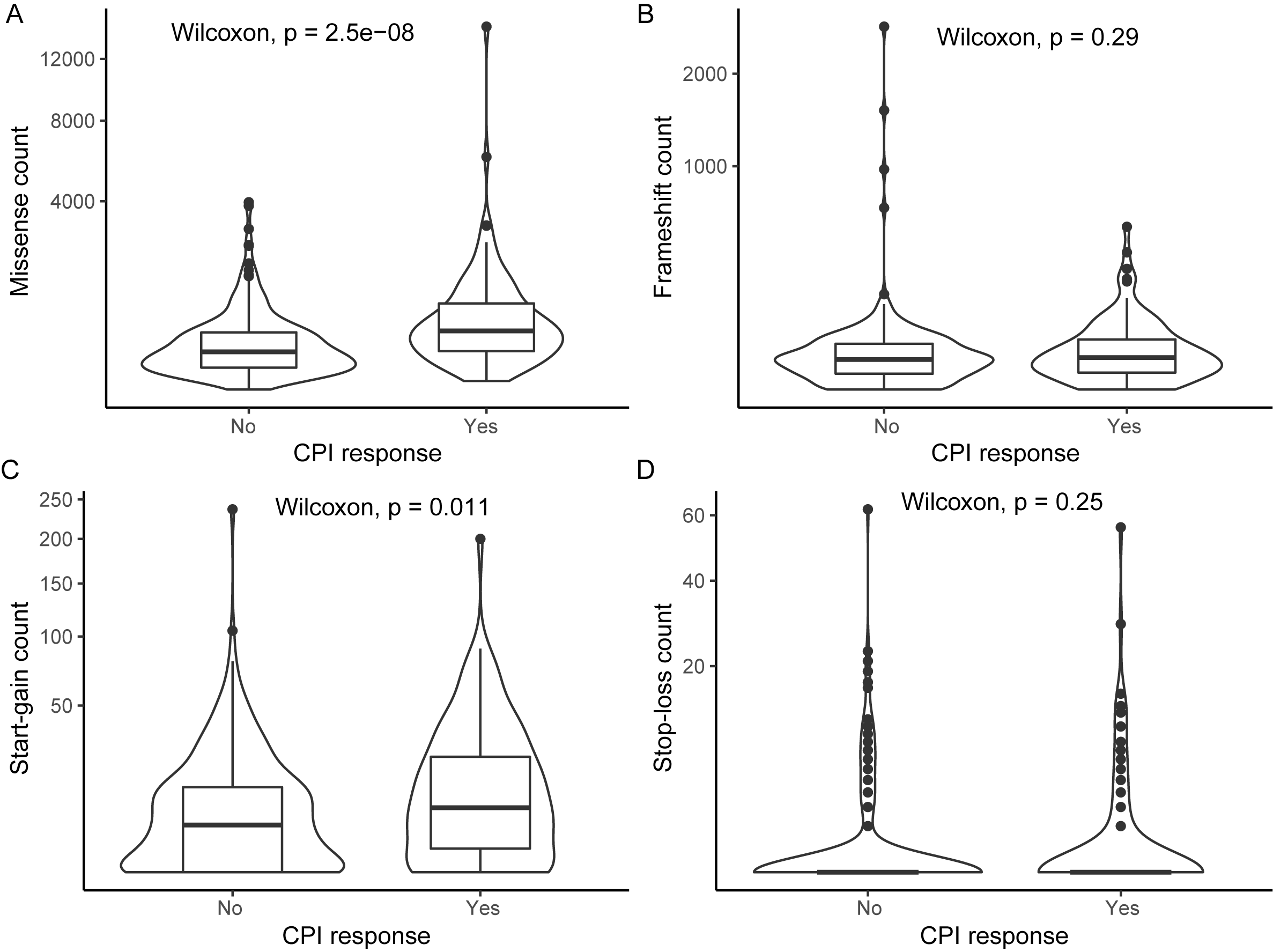

Supplement: Supplementary Figure 3 — Univariate analysis of CPI response based on (A) missense, (B) frameshift, (C) start-gain or (D) stop-loss neoantigen count. [file Image_3.tif]
